# Comparison of two strategies for initiating renal replacement therapy in the ICU: study protocol plan for a multicenter, randomized, controlled trial from the AKIKI research group

**DOI:** 10.1186/cc14379

**Published:** 2015-03-16

**Authors:** S Gaudry, D Hajage, F Schortgen, L Martin-Lefevre, J Ricard, D Dreyfuss

**Affiliations:** 1 Hôpital Louis Mourier, Colombes, France; 2 Hôpital Bichat, Paris, France; 3 Hôpital Henri Mondor, Créteil, France; 4 CHD La Roche sur Yon, France

## Introduction

There is currently no validated strategy for the timing of renal replacement therapy (RRT) for acute kidney injury (AKI) in the ICU when short-term life-threatening metabolic abnormalities are absent. No adequately powered prospective randomized study has to date addressed this issue. As a result, significant practice heterogeneity exists and may expose patients either to unnecessary hazardous procedures or to undue delay in RRT.

## Methods

This is a multicenter, prospective, randomized, open-label parallel-group clinical trial that compares the effect of two RRT initiation strategies on overall survival of critically ill patients receiving intravenous catecholamines and/or invasive mechanical ventilation and presenting with RIFLE F stage of AKI. In the early strategy, RRT is initiated immediately. In the delayed strategy, clinical and metabolic conditions are closely monitored and RRT is initiated only when one or more events (severity criteria) occur, including: oliguria or anuria for more than 72 hours after randomization, serum urea concentration >40 mmol/l, serum potassium concentration >6 mmol/l, serum potassium concentration >5.5 mmol/l persisting despite medical treatment, arterial blood pH <7.15 in a context of pure metabolic acidosis (PaCO_2_ <35 mmHg) or in a context of mixed acidosis with a PaCO_2_ >50 mmHg without possibility of increasing alveolar ventilation, acute pulmonary edema due to fluid overload despite diuretic therapy leading to severe hypoxemia requiring oxygen flow rate >5 l/minute to maintain SpO_2_ >95% or FiO_2_ >50% under invasive or non-invasive mechanical ventilation. The primary endpoint is overall survival, measured from randomization (D0) until death, regardless of the cause. The minimum follow-up duration for each patient will be 60 days. To demonstrate a 14% decrease in mortality, a total of 546 subjects (273 per group) should be randomized.

## Results

Enrollment is ongoing. After the first interim analysis, the DSMB recommended to continue the study. On 5 December 2014, 318 patients were included in the trial.

## Conclusion

The AKIKI study will be one of the very few large randomized controlled trials evaluating mortality according to the timing of RRT in critically ill patients with RIFLE F stage of AKI. Results should help clinicians better decide when to initiate RRT.

